# Identification of CaVβ1 Isoforms Required for Neuromuscular Junction Formation and Maintenance

**DOI:** 10.3390/cells14151210

**Published:** 2025-08-06

**Authors:** Amélie Vergnol, Aly Bourguiba, Stephanie Bauché, Massiré Traoré, Maxime Gelin, Christel Gentil, Sonia Pezet, Lucile Saillard, Pierre Meunier, Mégane Lemaitre, Julianne Perronnet, Frederic Tores, Candice Gautier, Zoheir Guesmia, Eric Allemand, Eric Batsché, France Pietri-Rouxel, Sestina Falcone

**Affiliations:** 1 Centre de Recherche en Myologie, Sorbonne Université, INSERM UMRS 974, Institut de Myologie, 75013 Paris, France; amelie.vergnol@orange.fr (A.V.); a.bourguiba@institut-myologie.org (A.B.); stephanie.godard-bauche@sorbonne-universite.fr (S.B.); m.traore@institut-myologie.org (M.T.); m.gelin@institut-myologie.org (M.G.); c.gentil@institut-myologie.org (C.G.); s.pezet@institut-myologie.org (S.P.); l.saillard@institut-myologie.org (L.S.); p.meunier@institut-myologie.org (P.M.);; 2 Phénotypage du Petit Animal—UM28 INSERM, Sorbonne Université, 75013 Paris, France; megane.lemaitre@sorbonne-universite.fr (M.L.); julianne.perronnet@sorbonne-universite.fr (J.P.); 3 Institut Imagine—BIP-D Plateforme de Bioinformatique, Université Paris Cité, 75015 Paris, France; frederic.tores@institutimagine.org; 4 Institut Imagine—U1163 INSERM, Université Paris Cité, 75015 Paris, France; candice.gautier.pro@gmail.com (C.G.); eric.allemand@inserm.fr (E.A.); 5 Institut de Biologie Paris-Seine (IBPS)—UMR 7238 CNRS, Sorbonne Université, 75005 Paris, France; eric.batsche@sorbonne-universite.fr

**Keywords:** CaVβ isoforms, neuromuscular junctions, skeletal muscle, Cacnb1, promoters, Long-read sequencing

## Abstract

Voltage-gated Ca^2+^ channels (VGCCs) are regulated by four CaVβ subunits (CaVβ1–CaVβ4), each showing specific expression patterns in excitable cells. While primarily known for regulating VGCC function, CaVβ proteins also have channel-independent roles, including gene expression modulation. Among these, CaVβ1 is expressed in skeletal muscle as multiple isoforms. The adult isoform, CaVβ1D, localizes at the triad and modulates CaV1 activity during Excitation–Contraction Coupling (ECC). In this study, we investigated the lesser-known embryonic/perinatal CaVβ1 isoforms and their roles in neuromuscular junction (NMJ) formation, maturation, and maintenance. We found that CaVβ1 isoform expression is developmentally regulated through differential promoter activation. Specifically, CaVβ1A is expressed in embryonic muscle and reactivated in denervated adult muscle, alongside the known CaVβ1E isoform. Nerve injury in adult muscle triggers a shift in promoter usage, resulting in re-expression of embryonic/perinatal Cacnb1A and Cacnb1E transcripts. Functional analyses using aneural agrin-induced AChR clustering on primary myotubes demonstrated that these isoforms contribute to NMJ formation. Additionally, their expression during early post-natal development is essential for NMJ maturation and long-term maintenance. These findings reveal previously unrecognized roles of CaVβ1 isoforms beyond VGCC regulation, highlighting their significance in neuromuscular system development and homeostasis.

## 1. Introduction

CaVβ1 proteins, encoded by the Cacnb1 gene from exon1 to exon14, exist as several variants in multiple tissues. In skeletal muscle, the Cacnb1D transcript, which spans exon1 to exon13end, produces CaVβ1D, the constitutive adult isoform. The Cacnb1E transcript, ranging from exon2B to exon14, encodes CaVβ1E, an embryonic isoform that is re-expressed in adult skeletal muscle after nerve damage [1]. CaVβ1 proteins were originally described as key modulators of the skeletal muscle voltage-gated calcium (Ca^2+^) channel (VGCC) CaV1 [2] and have been shown to be required for a proper Excitation–Contraction Coupling (ECC). In particular, CaVβ1D, which localizes at the triads as a subunit of CaV1.1, likely holds the regulatory function for this process in adult skeletal muscle, modulating the interaction between the two Ca^2+^ channels CaV1.1 and Ryanodine Receptor 1 (RyR1) [1]. In addition, CaVβ1 proteins have been reported as modulators of gene expression independently of the CaV1 channel, notably in muscle precursor cells regulating myogenesis, and in adult skeletal muscle after nerve damage, contributing to limiting muscle mass loss [1,3,4]. These roles are presumably accomplished by the embryonic CaVβ1E isoform, displaying nuclear localization [1,3,4].

In addition to the putative roles of Ca_V_β1D in regulating ECC at the triad and of Ca_V_β1E in modulating gene expression in the nucleus, another study highlighted the relevance of Ca_V_β1 proteins in skeletal muscle by showing their crucial implication in the nerve–muscle connection during embryogenesis [5]. During mammalian development and into early post-natal life, the formation, maturation, and stabilization of the neuromuscular junction (NMJ) are complex processes that require multiple cues for the establishment of functional nerve–muscle synapses. At early embryogenic stages, before the initiation of muscle innervation, and intrinsic to the muscle, both co-receptors LRP4 (low-density lipoprotein receptor-related protein 4) and MuSK (muscle-specific kinase) cluster together with clusters of aneural acetylcholine receptor (AChR) aggregates in a medial region of myotubes [6,7]. This phenomenon, known as muscle pre-patterning, represents the first initiating event of the NMJ formation [8]. Alterations in aneural AChR pre-patterning give rise to abnormal growth and spreading of innervating neurites along the muscle fibers [5]. Between E14 and E18, innervation by the motor neuron terminal drives the release of neural agrin, triggering the aggregation of AChR beneath the nerve terminal via Agrin/LRP4/MuSK signaling. Later, depolarization induces the release of acetylcholine (ACh) from the nerve ending, which activates the postsynaptic AChR essential for NMJ maturation. In addition to being fundamental for NMJ formation and maturation, agrin/LRP4/MuSK signaling is also critical for NMJ maintenance and function throughout life. Moreover, Docking protein 7 (Dok7), one of the key players in maintaining NMJ integrity, is indispensable for MuSK basal activity and activation of the tyrosine kinase domain by agrin [8,9].

The role of Ca^2+^ influx and release through CaV1.1 and RyR1 on AChR pre-patterning was investigated by using different models having ECC or Ca^2+^ alteration. It was observed that dysgenic (CaV1.1-null) mouse embryos, lacking voltage sensor as well as Ca^2+^ channel functions, display abnormal AChR distribution [10]. However, this distribution is unaffected in dyspedic (RyR1-/-) mouse embryos, keeping Cav1.1 Ca^2+^ channel activity, despite both CaV1.1-null and RyR1-/- mice having disrupted ECC. In addition, the genetically modified model of CaV1.1 (CaV1.1nc/nc), displaying preserved voltage sensor and altered Ca^2+^ channel functions, shows normal AChR pre-patterning. Finally, the double RyR1-/-; CaV1.1nc/nc KO embryos, which lack both Ca^2+^ influx from CaV1.1 and Ca^2+^ release from RyR1, exhibit abnormal AChR distribution [11]. These data demonstrate that Ca^2+^ influx and release through Ca_V_1.1 and RyR1, rather than the ECC mechanism itself, are crucial for AChR aggregation during NMJ formation.

Similar to these models, Cacnb1^−/−^ mice show severely affected NMJ pre-patterning, independently of ECC [5]. However, the role of Ca_V_β1 proteins in modulating various other aspects and mechanisms of NMJ formation, maturation, and maintenance may have been underestimated.

The importance of Ca^2+^ influx/release rather than ECC itself was further demonstrated while the double RyR1^−/−^; Ca_V_1.1^nc/nc^ KO model, which lacks both Ca^2+^ influx from Ca_V_1.1 and release from RyR1, shows an abnormal AChR distribution [11].

In the present study, we aimed at identifying the major representative Cacnb1 transcripts in embryonic pre- and post-innervated muscles in order to characterize their function in the formation and maturation of NMJ, as well as for the NMJ maintenance implication in adult innervated muscles. We confirmed the expression of Cacnb1D in adult innervated muscle and found that Cacnb1A, in addition to Cacnb1E, is expressed in embryonic and denervated adult mouse skeletal muscle. We demonstrated that the expression of the different *Cacnb1* isoforms is driven by the activation of specific promoters on embryonic and adult muscles, and that denervation is associated with the restoration of the embryonic epigenetic program. Analysis of the function of embryonic isoforms in the formation and maturation of NMJs after ablation of the embryonic Ca_V_β1 isoforms revealed a precocious differentiation of primary myotubes in vitro, associated with an increase in the size of neural agrin-triggered AChR aggregates, while it induced a decrease in the size of AChR areas in early post-natal myofibers. In adult innervated muscle, Ca_V_β1 embryonic isoform ablation led to NMJ destabilization, accompanied by denervation-like signs, accordingly to what was observed in the early phases of muscle aging [12].

Overall, our findings reveal the crucial relevance of Ca_V_β1 embryonic isoforms in processes needed for the establishment, maturation, and stability of functional NMJ, shedding light on novel fundamental players involved in the development, growth, and maintenance of the neuromuscular system.

## 2. Materials and Methods

### 2.1. Plasmids and Adeno-Associated Virus (AAV) Production

pSMD2-shEx2A was previously reported [1] to specifically target *Cacnb1* exon2A (target sequence CCTCGGATACAACATCCAACA), therefore downregulating *Cacnb1* embryonic isoforms specifically. pSMD2-shEx2A was prepared in an AAV1 serotype vector by the AAV production facility of the Center of Research in Myology via transfection in 293 cells as previously described [1]. The final viral preparations were kept in phosphate-buffered saline (PBS) solution at −80 °C. The particle titer (number of viral genomes: vg) was determined by quantitative PCR. Transduction of this AAV1-containing plasmid will be referred to as shEx2A treatment.

### 2.2. Animals and Ethics

All animals were housed under specific-pathogen-free (SPF) conditions, with a 12-h light cycle with access to rodent food and water ad libitum. Embryos were from breeding housed directly in the UMS28 animal facility or from gestating females from Janvier Lab. Newborn (7-day-old) male and female mice were from breeding housed directly in the UMS28 animal facility. Adult (2 months old at the beginning of the procedures) C57/BL6 female mice were from Janvier Lab and accustomed to the animal facility for one week before undergoing the experimental procedures. At the end of the procedures, 7-day-old mice were euthanized by decapitation, while adult mice were euthanized by cervical dislocation.

Ethics: Animal experimental procedures were performed in accordance with national and European guidelines for animal experimentation, with the approval of the institutional ethics committee. Ethic Committee Name: MENESR (MINISTERE DE L’ENSEIGNEMENT SUPERIEUR, DE LA RECHERCHE ET DE L’INNOVATION, “Ministry of Higher Education and Research”).

Approval Code: #17975 (denervation procedures) and #45606 (AAV ShE2A injection)

Approval Date: #19975: 2020/31/12; #45606 2024/13/05 (MENESR: Project #17975 and Project #45606).

### 2.3. Injection Procedures

For the experiment on adult mice, 8-week-old female animals were injected with AAV1-shEx2A or AAV1-scramble in the Tibialis Anterior (TA) muscles. Anesthesia was achieved using isoflurane (3% induction, 2% maintenance), and analgesia was achieved using buprenorphine (Vetergesic 1mg/kg, subcutaneous). One intramuscular injection (40 µL/TA or 100 µL/gastrocnemius (GAS) + Soleus (Sol)) was performed at a final titer of 1.10^9^ vg/mg of muscle. Mice were sacrificed 8 weeks after the injection. For experiments on early-stage muscle development, animals (female and male) were injected at 7 days old with AAV1-shEx2A or AAV1-scramble in TA and GAS/Sol muscles. Anesthesia was achieved using isoflurane (4% induction, 4% maintenance). One intramuscular injection (10 μL/TA or 15 µL/GAS+Sol) was performed at a final titer of 1.10^9^ vg/mg of muscle. Mice were sacrificed 3 weeks after the injection. Muscles were collected and either fixed in paraformaldehyde (PFA) 4% or frozen in isopentane precooled in liquid nitrogen and stored at −80 °C until histology or molecular analysis.

### 2.4. Denervation Procedures

The right sciatic nerves were neuroectomized (ablation of a 5 mm segment of the sciatic nerve) under general anesthesia (isoflurane: 3% induction, 2% maintenance) and under buprenorphine (Vetergesic 1mg/kg, subcutaneous). Mice were sacrificed 2, 3, or 14 days after denervation, and muscles were collected and either fixed in PFA 4% or frozen in isopentane precooled in liquid nitrogen and stored at −80 °C until histology or molecular analysis.

### 2.5. Functional Analyses

The force measurement of TA was evaluated by measuring in vivo muscle contraction in response to nerve stimulation, as previously described [1]. Mice were anesthetized with isoflurane, 3%, the knee and paw were fixed in place, and the distal tendon of the muscle was attached to the lever arm of a servomotor system (305B, Dual-Mode Lever, Aurora Scientific, Aurora, ON, Canada) using a silk ligature. Data were analyzed using the PowerLab system (4SP, ADInstruments) and software Labchart Pro v8.1 (ADInstruments, Colorado Springs, CO, USA). The sciatic nerve was stimulated using supramaximal square-wave pulses of 0.1 ms in duration. Capacity for force generation was evaluated by measuring the absolute maximal force that was generated during isometric contractions in response to electrical stimulation (frequency of 75–150 Hz; train of stimulation of 500 ms). Maximal isometric force was determined at L0 (the length at which maximal tension was obtained during the tetanus). Force was normalized by muscle mass as an estimate of specific force.

For the electroneuromyogram (ENMG), two monopolar reference and recording electrodes were inserted: one close to the patella tendon and the other in the middle of the TA muscle. A monopolar ground electrode was also inserted into the contralateral hindlimb muscle. The sciatic nerve was stimulated with a series of 10 stimuli at 5, 10, 20, and 40 Hz. Compound muscle activity potential (CMAP) was amplified (BioAmp, ADInstruments, Colorado Springs, CO, USA), acquired with a sampling rate of 100 kHz, and filtered with a 5 kHz low-pass filter and a 1 Hz high-pass filter (PowerLab 4/25, ADInstruments), and peak-to-peak amplitudes were analyzed with Labchart Pro v8.1.30 software (ADInstruments).

During all experiments, the mouse body temperature was maintained at 37 °C with radiant heat.

### 2.6. Primary Cells, Transduction, and High Differentiation Induction

Primary myoblasts from WT newborn mice were prepared as previously described [13]. Briefly, after hind limb muscle isolation, muscles were minced and digested for 1.5 h in PBS containing 0.5 mg/mL collagenase (Sigma, St. Louis, MO, USA) and 3.5 mg/mL dispase (Gibco, Waltham, MA, USA). The cell suspension was filtered through a 40 μm cell strainer and preplated in IMEM + 10% FBS + 0.1% gentamycin for 4 h to discard the majority of fibroblasts and contaminating cells. Non-adherent myogenic cells were collected and plated in IMDM + 20% FBS + 1% Chick Embryo Extract (MP Biomedical, Irvine, CA, USA) + 0.1% gentamycin in a 12-well plate or fluorodishes coated with IMDM 1:100 Matrigel Reduced Factor (BD Biosciences, San Diego, CA, USA). Differentiation was triggered by medium switch in IMDM + 2% horse serum + 0.1% gentamycin. A thick layer of Matrigel Reduced Factor (1:3 in IMDM) was added 24 h after differentiation medium addition. Myotubes were treated with 100 µg/mL of agrin, and the medium was changed every 2 days for 8 days of differentiation [13]. Preliminary experiments were performed to determine the Multiplicity of Infection (MOI) of AAV-shEx2A in muscle cells and assessed 5.10^5^ vg/cell to obtain the optimal silencing of embryonic Ca_V_β1 isoforms. AAV transduction was achieved concurrently with the induction of differentiation of primary muscle cells.

### 2.7. RNA Isolation and Gene Expression Analyses

Total RNA was extracted from muscle cryosections using Trizol (ThermoFisher Scientific, Waltham, MA, USA) and Direct-zol RNA MiniPrep w/Zymo-Spin IIC Columns (Ozyme, Saint-Cyr-l’École, France) and from cells using NucleoSpin RNA Columns (Macherey-Nagel, Allentown, PA, USA). An amount of 200 ng of total RNA was subjected to reverse transcription (RT) using the Superscript II Reverse transcriptase kit (18064022, ThermoFisher Scientific) or 1 µg of total RNA using Maxima H Minus Reverse Transcriptase (EP0753, ThermoFisher Scientific), with a mix of random primers (48190011, ThermoFisher Scientific) and oligo(dT) (18418020, ThermoFisher Scientific) to generate complementary DNA (cDNA). An amount of 2 µL of cDNA was amplified in 20 µL reactions in PCR Master Mix (M7505, Promega, Madison, WI, USA), Phusion High-Fidelity PCR Master Mix with GC Buffer (F532L, ThermoFisher Scientific), or Q5 Hot Start High-Fidelity 2X Master Mix (M0494S, New England Biolabs, Ipswich, MA, USA) for RT-PCR and RT-PCR triplex. An amount of 2 µL of 1:5 diluted cDNA was amplified in 10 µL reactions in Power SYBR Green PCR MasterMix (ThermoFisher Scientific) to perform quantitative real-time PCR (qPCR) on a StepOne Plus Real-Time PCR System (Applied Biosystems, Foster City, CA, USA). Data were analyzed using the ΔΔCT method and normalized to RPLPO (ribosomal protein lateral stalk subunit P0) mRNA expression. The sample reference to calculate mRNA fold change is indicated in each panel. Primers are listed in Table S1.

### 2.8. Nanopore Sequencing

An amount of 600 ng of total RNA was subjected to RT with an anchored poly-dT primer (12577011, ThermoFisher Scientific) and Maxima H Minus Reverse Transcriptase. The amplification of the Cacnb1 transcriptome was independently processed in each sample through two PCR steps, using 2 μL of diluted RT reactions (12-fold dilution). First, a pre-amplification was performed for 20 cycles using specific primers fused with universal sequences U1 and U2 (referenced in Table S2). These primers are specifically base-paired with the first and last exons of Cacnb1 transcripts. The PCR reactions were treated with exonuclease I to remove primer excess and purified with the NucleoMag^®^ NGS Clean-up and Size Select (Macherey-Nagel, Allentown, PA, USA). Next, a second amplification of 18 cycles was performed to incorporate barcodes associated with individual samples. All samples were combined to create a stoichiometric multiplexed library, which was prepared using the Oxford Nanopore SQK-LSK109 kit. The library was subsequently sequenced using a MinION Flow Cell (R9.4.1). The Oxford Nanopore data were analyzed as previously described [14], and Fastq files generated in this study were deposited in the ENA database (https://www.ebi.ac.uk/ena/browser) under accession code PRJEB89914 date access 12 June 2025.

### 2.9. ChIP Experiments

TA and GAS muscles were harvested from 8-week-old female mice, either innervated or 2 days after denervation. Tissues were finely minced with a scalpel in PBS, incubated for 10 min in PBS containing 1% formaldehyde, and then 5 min in PBS containing 125 mM glycine. Aliquots used for RNAPII immunoprecipitation were fixed a second time with disuccinimidyl glutarate (DSG) diluted to 2 mM in PBS for 40 min at room temperature, as previously described [14]. Tissues were then dissociated with a MACS dissociator using the muscle-specific program.

Resuspended material was incubated on ice for 5 min in 1 mL of chilled buffer A (0.25% Triton, 10 mM TRIS pH8, 10 mM EDTA, and 0.5 mM EGTA), for 30 min in 1 mL of buffer B (250 mM NaCl, 50 mM TRIS pH8, 1 mM EDTA, and 0.5mM EGTA), and then resuspended in 100 to 200 µL of buffer C (1% SDS, 10 mM TRIS pH8, 1 mM EDTA, and 0.5 mM EGTA) at room temperature. All buffers were extemporaneously supplemented by complete protease and PhosSTOP phosphatase inhibitors (Roche, Indianapolis, IN, USA). The cell suspensions in 0.6 mL µtubes were sonicated in a water bath at 4 °C for 15 min (15 s ON, 15 s OFF) with a Bioruptor apparatus (Diagenode) set on high power and then clarified by 10 min of centrifugation at 10,000 rpm, 4 °C. Shearing of the DNA was checked after reversing the crosslinking on agarose gel electrophoresis to be around 300–500 bp. Sheared chromatin was quantified by optical density (260 nm) and diluted 10-fold in IP buffer to the following final concentrations: 1% Triton, 0.1% NaDeoxycholate, 0.1% SDS, 150 mM NaCl, 10 mM TRIS pH8, 1 mM EDTA, 0.5 mM EGTA, and 1X protease and phosphatase inhibitors. Amounts of 15 µg of chromatin and 2 µg of antibodies were incubated in a final volume of 500 µL at 4 °C for 16 h on a wheel. An amount of 25 µL of saturated magnetic beads coupled to protein G (Dynabeads, Carlsbad, CA, USA) was used to recover the immuno-complexes. After 2 h of incubation, the bound complexes were washed extensively for 5 min at room temperature on a wheel in the following wash buffers: WBI (1% Triton, 0.1% NaDOC, 150 mM NaCl, and 10 mM TRIS pH8), WBII (1% Triton, 1% NaDOC, 150 mM KCl, and 10 mM TRIS pH8), WBIII (0.5% Triton, 0.1% NaDOC, 100 mM NaCl, and 10 mM TRIS pH8), WBIV (0.5% Igepal CA630 (Sigma, St. Louis, MO, USA), 0.5% NaDOC, 250 mM LiCl, 10 mM TRIS pH8, and 1 mM EDTA), WBV (0.1% Igepal, 150 mM NaCl, 20 mM TRIS pH8, and 1 mM EDTA), and WBVI (0.001% Igepal, 10 mM TRIS pH8). An amount of 20 µg of sheared chromatin was used as input, and ChIP beads were then boiled for 10 min in 100 µL of H_2_O containing 10% (V/W) chelex resin (BioRad, Hercules, CA, USA), followed by Proteinase K (0.5 mg/mL) digestion for 30 min at 55 °C, and then finally incubated 10 min at 100 °C. An amount of 1 µL of ChIP eluate was used for qPCR assays in 10 μL reactions with Brilliant III Ultra-Fast SYBR-Green Mix (Agilent, Santa Clara, CA, USA) using a Stratagene MX3005p system. The analysis of qPCR was performed using the MxPro QPCR software v4.1D version. Primers are listed in Table S1.

### 2.10. Immunoblotting

Approximately 500 µm of muscle cryosections from frozen muscle was homogenized with a Dounce homogenizer protein extraction buffer containing 50 mM Tris-HCl, pH 7.4, 100 mM NaCl, 0,5% NP40, with Halt Protease and Phosphatase inhibitor cocktail (78440, ThermoFisher Scientific). The samples were then centrifuged for 10 min at 1500× *g* at 4 °C, and then the supernatants were collected. Protein concentration was determined by Bradford assay (ThermoFisher Scientific). The samples were denatured at room temperature for 30 min (Ca_V_β1 proteins) or at 95 °C for 5 min (all other proteins), with Laemmli buffer with 5% βmercaptoethanol. Proteins were separated by electrophoresis (Nu-PAGE 4–12% Bis-Tris gel; Life Technologies, Carlsbad, CA, USA) and then transferred to nitrocellulose membranes (GE Healthcare, Chicago, IL, USA). Membranes were blocked with 5% BSA (CaVβ1 proteins) or 5% milk (all other proteins) and incubated overnight at 4 °C with primary antibodies (listed in Table S3) diluted in the blocking buffer. Membranes were then labelled with fluorescent secondary antibodies (listed in Table S4) diluted in blocking buffer for 1 h in the dark. Images were acquired with the LAS4000 camera (GE Healthcare, Chicago, IL, USA). Western Blot image analysis was performed with the public domain software ImageJ (analyze gel tool).

### 2.11. Immunofluorescence and Image Acquisition—Muscle Cryosections

Immunofluorescence procedures were performed on 10 µm muscle cryosections fixed on glass slides and stored at −80 °C. The slides were rehydrated in PBS, fixed with PFA 4% for 10 min, permeabilized with 0.5% Triton X-100 (Sigma-Aldrich, St. Louis, MO, USA), and blocked in PBS with 4% bovine serum albumin and 0.1% Triton X-100 for 1 h. Sections were incubated in PBS with 2% BSA and 0.1% Triton X-100 with primary antibodies (listed in Table S3) overnight at 4 °C.

Aspecific sites were blocked with BSA 5% 1 h, and slides were incubated for 1 h with secondary antibodies (listed in Table S4) and incubated 5 min with 4′,6′-diamidino-2-phenylindole (DAPI) for nuclear staining and mounted in Fluoromount (Southern Biotech, Birmingham, AL, USA).

### 2.12. Immunofluorescence and Image Acquisition—Muscle Fibers

TA muscles were collected, rinsed once in PBS, and fixed in a PBS 4% PFA solution at room temperature for 1 h. Groups of approximately 10 muscle fibers were gently dissected and incubated overnight in PBS with 0.1 M glycine at 4 °C with mild shaking. The next day, after a day of washing in PBS 0.5% and Triton X-100, fibers were incubated, permeabilized, and blocked for 6 h in PBS 3%, BSA 5%, goat serum, and 1% Triton X-100 at room temperature. Fibers were incubated with primary antibodies (Table S3) at 4 °C for 48 h in blocking solution. Fibers were then rinsed for several hours at room temperature, in PBS 0.5% and Triton X-100, and incubated overnight at 4 °C with secondary antibodies (Table S4) and α-Bungarotoxin Alexa Fluor conjugated-594 (Table S3) in blocking solution. Finally, after a day of washing in PBS 0.5% and Triton X-100, fibers were mounted on slides in non-hardening Vectashield mounting medium (H-1000, Vector Laboratories, Newark, CA, USA).

NMJ images were acquired with a Zeiss Axio Observer microscope coupled with an apotome module (63x objective) and edited with Zeiss Zen Lite 3.7 software. The same laser power and parameter settings were used throughout to ensure comparability. The images presented were single projected images obtained by overlaying sets of collected z-stacks. NMJ morphometric analysis was performed on confocal z-stack projections of individual NMJs by using an ImageJ-based workflow adapted from NMJ-morph [12]. At least 100 NMJs were analyzed per condition. NMJ morphometric analysis was performed in a blinded manner by the same investigator.

### 2.13. Immunofluorescence and Image Acquisition—Primary Myotubes

For immunofluorescence analyses, primary myotubes were seeded and cultured in fluorodishes (Dutscher, Bernolsheim, France). The cells were fixed in 4% PFA for 10 min and then permeabilized with PBS 5% Triton X-100. Aspecific sites were blocked with BSA 1% and goat serum 10% for 30 min. The cells were incubated with primary antibodies (listed in Table S3) overnight at 4 °C in PBS 0.1% saponin 1% BSA. The cells were washed three times and then incubated with secondary antibodies (listed in Table S4) and DAPI (1:10,000) for 1 h in the dark.

Fluorescence images of myotubes were acquired on a Spinning Disk confocal scanner unit (CSU-W1 YOKOGAWA, Musashino, Japan) with a 40x oil-immersion objective (plan fluor 40x/1.3 oil OFN25 DIC N2 cyan), coupled with a Photometrics PRIM 95B Camera. Software used was MetaMorph 7.10.2. Image analyses and quantification of AChR cluster size and distribution were performed with the public domain ImageJ software 1.54m version by using a macro developed in the lab by the MyoImage facility.

### 2.14. Statistical Analyses

For comparison between two groups, data were tested for normality using a Shapiro–Wilk test and for homoscedasticity using a Bartlett test, followed by parametric (two-tailed paired and unpaired Student’s *t*-tests) or non-parametric test (Mann–Whitney) to calculate *p* values. For comparison among more than two groups, according to normality, ordinary one-way ANOVA or Kruskal–Wallis tests were performed. According to homoscedasticity, Brown–Forsythe ANOVA or ANOVA was performed. When it was necessary, two-way ANOVA tests were performed to compare between groups

All ANOVA and Kruskal–Wallis tests were followed by appropriate post hoc tests. All statistical analyses were performed with GraphPad Prism 8.4.3 or 9.0.0 software (Dotmatics, Boston, MA, USA), with statistical significance set at *p* < 0.05.

## 3. Results

### 3.1. Identification of a Novel Ca_V_β1 Isoform in Adult Denervated and Embryonic Skeletal Muscles

**Figure 1:**
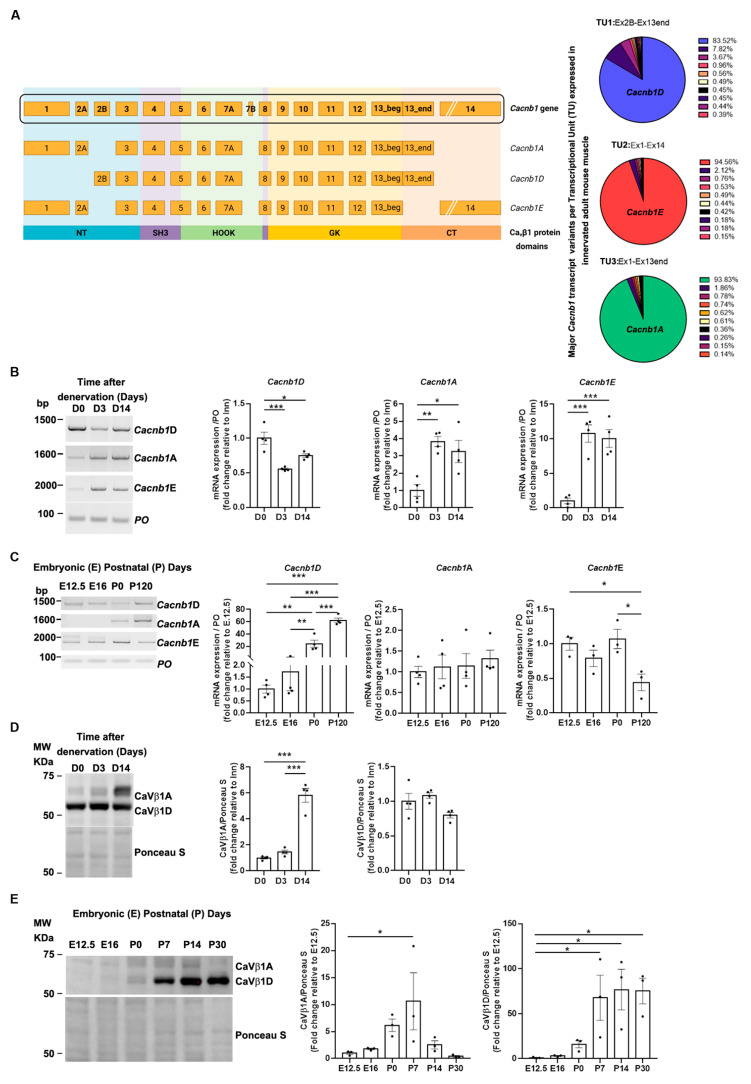
Identification of a new Ca_V_β1 isoform in embryonic muscles. (**A**) *Cacnb1* gene and skeletal muscle transcript variants, Cacnb1A, Cacnb1D, and Cacnb1E, as well as the corresponding protein domains, are represented. Full-length sequencing of the Cacnb1 transcript through Nanopore technology led to the identification of one major isoform per TU. (**B**) RT-PCR and quantification of the three full-length *Cacnb1* variants in innervated (D0) TA muscles or after 3 (D3) and 14 days of denervation. PO was the loading control. (**C**) Western Blot and quantification of Ca_V_β1A and Ca_V_β1D in embryonic (E) and post-natal (P) days. Ponceau S was the loading control. (**D**) Western Blot and quantification of Ca_V_β1A and Ca_V_β1D after 3 (D3) and 14 days of denervation. (**E**) Western Blot and quantification of Ca_V_β1A and Ca_V_β1D in embryonic (E) muscles and adult (P) TA muscles. Ponceau S was the loading control. All data are mean ± SEM (* *p* < 0.05, ** *p* < 0.01, and *** *p* < 0.001). *p* values were calculated by ordinary one-way ANOVA followed by (**B**–**D**) Tukey’s and (**E**) Dunnett’s multiple comparison tests.

The first description of the *Cacnb1E* embryonic isoform emerged from RT-PCR and genome-wide transcriptomic analyses [1]. This study revealed modulation of *Cacnb1* expression at the exon level. However, the existence of several predicted though unidentified isoforms sharing the same exons adds complexity to the interpretation of the results. To address this issue and go deeper in this characterization, we delineated the landscape of full-length Cacnb1 transcripts in adult innervated and denervated mouse skeletal muscle using targeted Oxford Nanopore sequencing [15,16] (Tibialis Anterior: TA; Gastrocnemius: GAS) in two conditions in which modulation of the expression of *Cacnb1* isoforms was previously observed [1] (Figure 1A; Figure S1). The visualization of the high-quality aligned Nanopore reads revealed the vast diversity of *Cacnb1* transcript variants, but with one major isoform per transcription unit (TU): more than 94% of the transcripts of the TU#1, ranging from exon1 to exon14, correspond to *Cacnb1E* isoform and more than 83% of the transcripts of the TU#2, ranging from exon2B to exon13end, correspond to *Cacnb1D* isoform. The examination of the TU#3, ranging from exon1 to exon13end, unveiled that more than 93% of the transcripts of this TU correspond to *Cacnb1A* (Figure 1A; Figure S1). It should be noted that these percentages represent the predominance of each isoform within its respective transcriptional units, not the overall abundance in the adult muscle. We validated these data by performing RT-PCR analyses of full-length transcripts in adult innervated and denervated as well as embryonic and perinatal limb mouse muscles [1]. We confirmed that these three transcripts were expressed in adult mouse muscle and that the expression of both *Cacnb1A* and *Cacnb1E* significantly increased in denervated muscle (*Cacnb1A*: *p* = 0.0041; *Cacnb1E:* *p* = 0.0001), while *Cacnb1D* decreased (Figure 1B). By amplifying full-length transcripts of *Cacnb1A*, *E*, and *D* from embryonic (E), newborn (P0), and adult (P120) mouse muscle extracts, we observed the appearance of *Cacnb1D* at P0 to be fully expressed in adulthood, as expected (Figure 1C). Additionally, both *Cacnb1A* and *E* transcripts were expressed during embryogenesis and adulthood (Figure 1C). We analyzed protein expression of Ca_V_β1 isoforms by Western Blot using a custom antiserum antibody specific to Ca_V_β1D and A variants (epitope coded by RNA sequence in exon13end). However, for Ca_V_β1E, no specific antibody for this isoform could be found, neither customized nor commercially available. In adult muscle, the levels of a 58kDa band, corresponding to Ca_V_β1A, increased in denervated muscle, while the 53kDa band intensity, corresponding to the Ca_V_β1D, did not change in this condition (Figure 1D). Wondering about the expression kinetics of Ca_V_β1D and Ca_V_β1A variants at various age stages, we measured their levels in embryonic muscles at E12.5 and E16, and at post-natal days P0, -7, -14, and -30 by using the same custom antibody. We confirmed that Ca_V_βA appeared in E16 until P14 and became weakly expressed in P30 innervated muscle, whereas Ca_V_βD started at P0 to be stably expressed in adult muscle (Figure 1E).

Overall, our results reveal that Cacnb1A, and not only Cacnb1E, transcripts are expressed in mouse skeletal muscle during embryogenesis until first post-natal life and increase after nerve damage, with Ca_V_β1A protein following this pattern, whereas Ca_V_β1E, due to the demission of previously commercialized antibodies, cannot be detected at the protein level. In addition, we validate the expression of the Cacnb1D transcript and the corresponding Ca_V_β1D protein exclusively after birth. Taken together, these data underline the complexity of Ca_V_β1 isoforms and intrigue about the specific role of each of these Ca_V_β1 proteins in skeletal muscle physiology.

### 3.2. Activation of Distinct Promoters for Cacnb1 Isoform Expression in Embryonic and Adult Muscles

**Figure 2:**
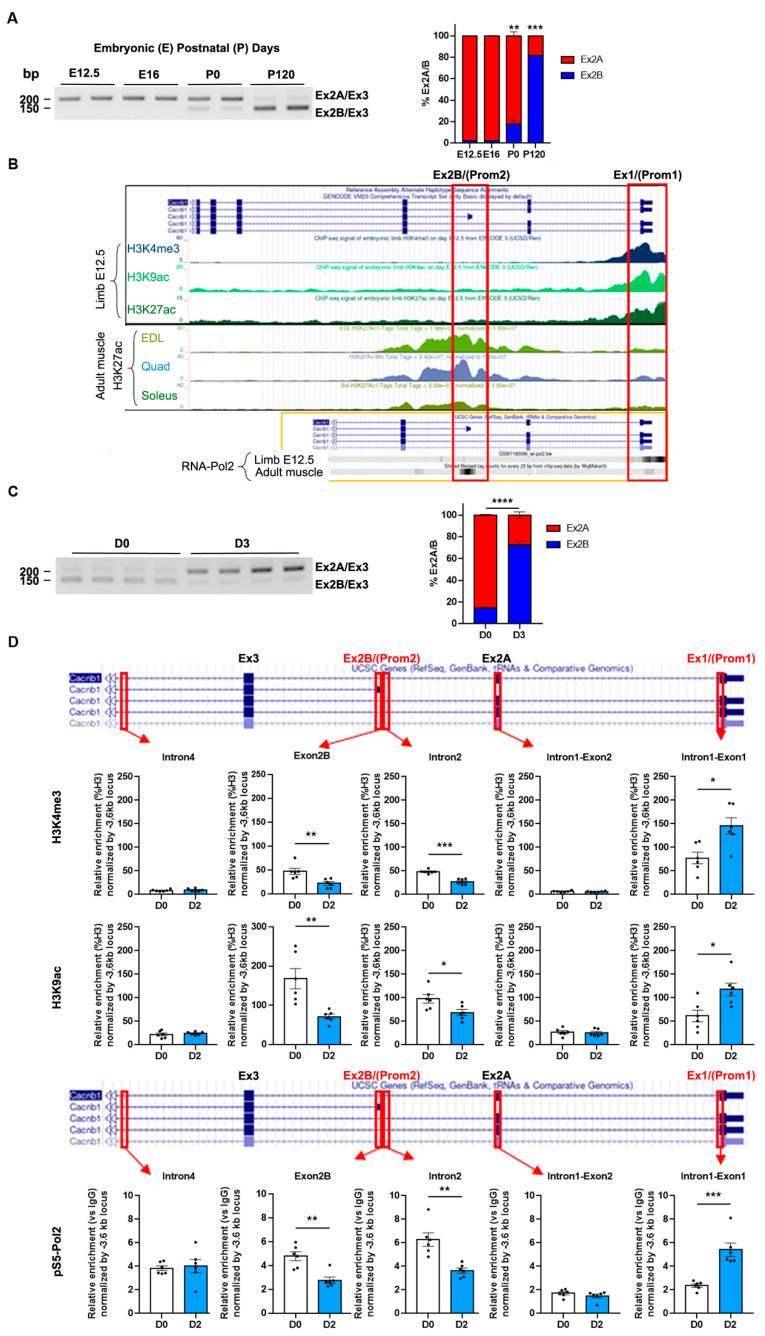
Two specific and distinct promoters drive the expression of *Cacnb1* isoforms in skeletal muscle. (**A**) Triplex RT-PCR and quantification of the percentage of exon2A versus exon2B inclusion in embryonic (E) and adult (P) TA muscles. (**B**) Visualization of epigenetic marks and RNA Polymerase II at *Cacnb1* promoters using UCSC Genome Browser. Image displaying the location of exon2B (Ex2B) containing the promoter site 2 (Prom2), exon1 (Ex1) containing the promoter site 1 (Prom1), activating epigenetic marks (H3K3me3, H3K9ac, and H3K27ac), and RNA Polymerase II occupancy at Prom1 and Prom2 in either embryonic (E12.5) muscles (ENCODE project) or adult muscles (Extensor digitorum longus (EDL), Quadriceps, and Soleus). (**C**) Triplex RT-PCR and quantification of the percentage of exon2A versus exon2B inclusion in innervated and 3-day denervated adult TA muscles (D3). (**D**) Chromatin immunoprecipitation (ChIP) followed by PCR analysis showing the presence of activating epigenetic marks (H3K4me3 and H3K9ac) and Serine5-phosphorylated RNA Polymerase II (pS5-Pol2) occupancy at Prom1 and Prom2 in innervated and 2-day denervated adult TA/Gas muscles. All data are mean ± SEM (* *p* < 0.05, ** *p* < 0.01, *** *p* < 0.001, and **** *p* < 0.0001). *p* values were calculated by ordinary two-way ANOVA followed by Tukey’s multiple comparison test (**A**), and Sidak’s multiple comparison test (**C**),or unpaired *t*-test (**D**).

To further investigate the transition of embryonic versus adult Ca_V_β1 isoforms, we performed triplex RT-PCR analyses to decipher the kinetics of exon2B inclusion that characterizes the adult Cacnb1D isoform, versus the kinetics of exon2A exclusion for identifying the embryonic Cacnb1A/E variants. As expected, we observed that exon2A was present from E12.5, while exon2B expression increased at birth and was highly expressed in adult muscle (Figure 2A). We then wondered if Cacnb1D and Cacnb1A/E expressions might be orchestrated by the activation of distinct promoters in embryonic and adult muscles. Nanopore sequencing revealed that the mRNAs starting at exon1 were never associated with exon2B (Figure S1). This suggests that the expression of exon2B may be exclusively triggered by a promoter located within this exon. To validate this hypothesis, we performed an in silico analysis (ENCODE project, UCSC Genome browser) of ChIPseq signals of methylation and acetylation histone marks and RNA-Polymerase II occupancy on the mouse *Cacnb1* gene in embryonic muscles compared to adult hind limb muscles. We found that methylation marks H3K4me3, H3K9ac, and H3K27ac, characteristic of active promoters [17], were mostly located at exon1 in E12.5 mouse muscle, while H3K27ac was mainly located at exon2B in three different adult mouse hind limb muscles (Figure 2B). In addition, RNA-Polymerase II was recruited at *Cacnb1* exon1 in E12.5 mouse muscle and at exon2B in adult mouse muscle (Figure 2B), suggesting a transcription initiation site [18]. Together, these data strongly suggest that distinct and specific promoters are used for the expression of embryonic (Prom1) versus adult (Prom2) Cacnb1 isoforms. We then asked whether the innervation status in adult muscle could affect the activity of *Cacnb1* promoters, therefore modulating the expression of Cacnb1 isoforms. We found that the percentage of exon2B inclusion was higher in innervated and decreased in denervated muscles, whereas exon1 inclusion was very low in innervated and increased significantly in denervated muscle RNA extracts (Figure 2C). To evaluate the activity of the putative specific promoters, we performed a ChIP-PCR analysis on the *Cacnb1* gene. We observed a reduction in epigenetic marks of active promoters (H3K4me3 and H3K9ac) at the Prom2 site, while these marks were enriched around Prom1 following denervation. Accordingly, the quantification of RNA-Polymerase II recruitment displayed increased enzyme occupancy around Prom1 and decreased enzyme occupancy at the Prom2 site in denervated compared to innervated muscles (Figure 2D and Figure S2).

Taken together, our results decipher the *Cacnb1* isoform transition from embryonic to adult isoforms and show that its regulation is driven by the activation of two distinct and specific promoters. Furthermore, we demonstrate that adult skeletal muscle restores the embryonic epigenetic program in the *Cacnb1* gene after nerve damage, suggesting a role for *Cacnb1* in the mechanisms aiming at recovering or maintaining a proper innervation status and potentially in other events associated with neuromuscular physiology.

### 3.3. Role of Embryonic Ca_V_β1 Isoforms in AChR Clustering

The expression of different *Cacnb1* isoforms at embryonic and adult stages has raised the question of the specific role of different variants in the formation, maturation, and maintenance of the NMJ.

**Figure 3:**
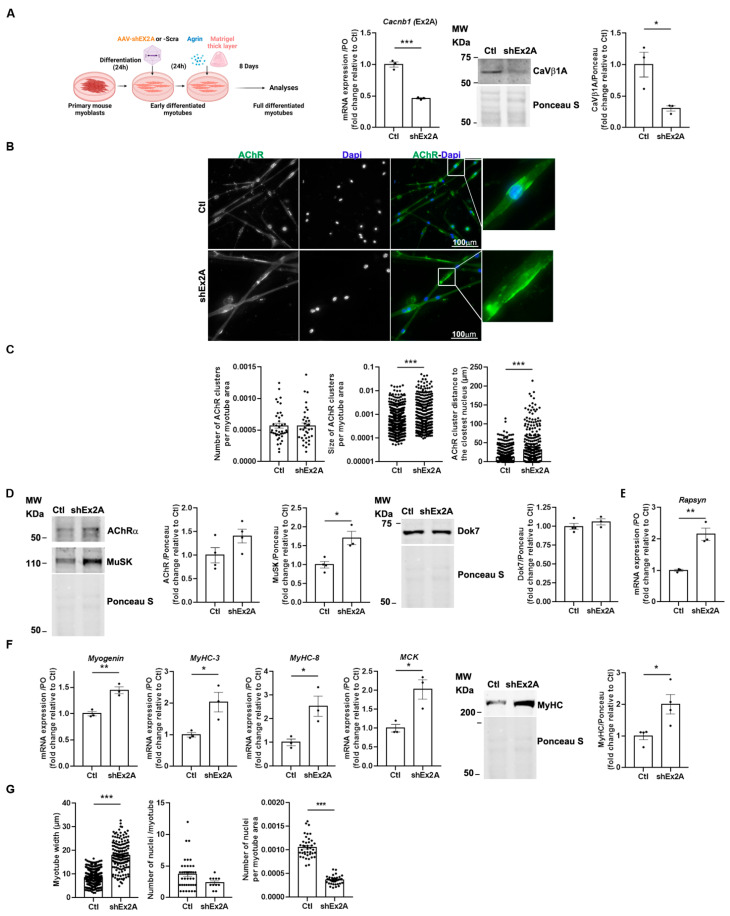
Downregulation of embryonic Ca_V_β1 isoforms induces bigger AChR aggregates and myotube precocious maturation in an in vitro model of highly differentiated myotubes. (**A**) Schematic representation of the experimental protocol. RT-qPCR of *Cacnb1* exon2A (Ex2A), Western Blot, and quantification of Ca_V_β1A in the control (Ctl) and shEx2-treated myotubes. (**B**) Immunofluorescence (IF) images of the control or shEx2A-treated myotubes stained with AChRa1 (green) and DAPI (blue). (**C**) Quantification of AChR cluster number and size per myotube area and distance between AChR clusters and the nearest nuclei. (**D**) Western Blot and quantification of AChR, MuSK, and Dok7 in the control and shEx2A-treated myotubes. (**E**) RT-qPCR of *Rapsyn* in the control and shEx2A-treated myotubes. (**F**) RT-qPCR of *Myogenin*, *MyHC-3*, *MyHC-8*, and *MCK* in the control and shEx2A-treated myotubes and Western Blot and quantification of MyHC in the control and shEx2A-treated myotubes. (**G**) Quantification of the number of nuclei per myotube and number of nuclei per myotube in the control and shEx2A-treated myotubes. All data are mean ± SEM (* *p* < 0.05, ** *p* < 0.01, and *** *p* < 0.001). *p* values were calculated by an unpaired *t*-test (**A**–**G**).

We investigated the role of embryonic *Cacnb1* isoforms in agrin-mediated AChR clustering in highly differentiated primary myotubes [13,19,20] by downregulating these isoforms using an adeno-associated virus 1 (AAV1) carrying a short hairpin targeting a sequence expressed in exon2A of *Cacnb1* pre-RNA, thus specifically silencing Ca_V_β1A and E transcripts and proteins (shEx2A) [1] (Figure 3A). After validating their downregulation (Figure 3A), we observed that their absence had no effect on AChR aggregation capacity, as shown by a similar number of AChR clusters per myofiber surface compared to the control condition (Figure 3B,C). However, it induced a significant rise in AChR cluster size, with a mean fold increase of 2.93 (Figure 3B,C). Additionally, we observed an augmented distance between AChR aggregates and the nearest nuclei, suggesting that embryonic Ca_V_β1 isoforms may play a role in positioning the future sub-synaptic nuclei (Figure 3B,C). To identify the mechanisms responsible for these in vitro AChR clustering abnormalities, we measured protein expression of AChRα as well as key players in aggregate formation/stabilization, including MuSK and Dok7 [9,21,22]. Although we did not observe a significant modification of AChRα and Dok7 protein levels, we found higher MuSK expression after silencing Ca_V_β1A and E compared to the control (Figure 3D). To understand if other molecules implicated in the stabilization of AChR clustering in aneural myotubes could also be impacted, we measured the gene expression level of Rapsyn [23,24]. We found increased expression of its transcript (Figure 3E), suggesting that the lack of embryonic CaβV1 isoforms could eventually affect AChR aggregates’ stability.

Then, we investigated the potential role of embryonic *Cacnb1* isoforms on the myogenic differentiation process. Despite enhanced Myogenin expression, we measured significantly higher MyHC-3, MyHC-8, and MCK mRNA expressions (*p* = 0.0318, *p* = 0.027, and *p* = 0.0203, respectively), as well as MyHC protein expression levels in shEx2A myotubes compared to the controls (Figure 3E). These data suggest that myogenic maturation was aberrantly accelerated in the absence of embryonic *Cacnb1* isoforms, corroborated by the finding that shEx2A-treated myotubes were wider compared to the controls (Figure 3F). To establish if myoblast fusion could be altered upon embryonic/perinatal Ca_V_β1 isoform downregulation, we also quantified the number of nuclei per myotube, and found no difference between the control and shEx2A-treated myotubes. However, the number of nuclei per total area of myotube decreased as a result of the treatment (Figure 3G), in accordance with the exacerbated myogenic maturation induced by the ablation of embryonic Cacnb1 isoforms. Altogether, these findings indicate that embryonic/perinatal Ca_V_β1 isoforms are not only needed to modulate the first phases of myogenesis as previously shown [3], but also to ensure the coordinated terminal muscle cell differentiation and AChR aggregation in response to agrin stimulation.

### 3.4. Role of Embryonic Ca_V_β1 Isoforms in Post-Natal NMJ Maturation/Maintenance

**Figure 4:**
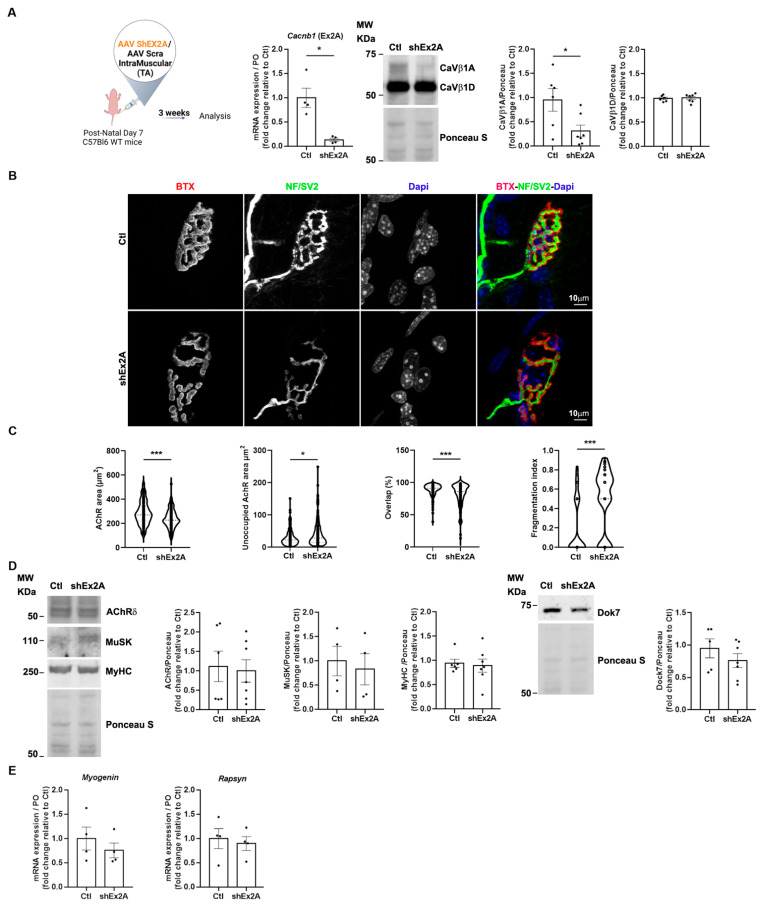
NMJ structural and molecular defects upon downregulation of embryonic Ca_V_β1 isoforms in TA muscles at post-natal stages. (**A**) Schematic representation of the experimental protocol. RT-qPCR of embryonic Cacnb1 variants using primer in exon2A in the control and shEx2-treated TA muscles, Western Blot, and quantification of Ca_V_β1A and Ca_V_β1D in muscles 3 weeks post-injection. (**B**) Immunofluorescence (IF) images of the control and shEx2A-treated TA muscles, 3 weeks post-injection, stained with α-bungarotoxin (BTX) for AChR (red), neurofilaments, and Synaptic Vesicle Glycoprotein 2 (NF/SV2) (green) and DAPI (blue). (**C**) Quantification of NMJ morphology with area of AChR, unoccupied AChR area, overlap between nerve terminals and postsynaptic apparatus, and fragmentation index. (**D**) Western Blot and quantification of AChRδ, MuSK, and MyHC in the control and shEx2A-treated TA muscles, 3 weeks post-injection. (**E**) RT-qPCR of Myogenin and Rapsyn in the control and shEx2A-treated muscles. All data are mean ± SEM (* *p* < 0.05, and *** *p* < 0.001). *p* values were calculated by paired (A—Cacnb1 RT-qPCR-, E) and unpaired *t*-tests (A—Ca_V_β1A and Ca_V_β1D WB-, C, D).

The presence of embryonic/perinatal Ca_V_β1 isoforms expressed until at least P14 (Figure 1E) suggests their possible involvement in NMJ post-natal maturation/maintenance. To decipher the role of these proteins in that process, we downregulated their expression using AAV-shEx2A injected in the hind limb muscles of 7-day-old pups (Figure 4A), and then analyzed NMJ morphology 21 days later (P30 mice), when endplates are usually mature [25]. We quantified NMJ morphological parameters and found that the AChR area was significantly reduced upon embryonic *Cacnb1* variant depletion compared to the control, while the size of the AChR area unoccupied by nerve endings was increased (Figure 4B,C). Additionally, the overlap between nerve terminals and postsynaptic apparatus was decreased, and a significant NMJ fragmentation was observed (Figure 4B,C). Although NMJ morphology appeared altered with pre- and postsynaptic abnormalities, we did not observe alterations in AChRα, MuSK, or Dok7 protein expression (Figure 4D) nor in Myogenin gene expression (Figure 4E). Importantly, the transcript level of Rapsyn was also not altered (Figure 4E), indicating that embryonic *Cacnb1* variants control different mechanisms and/or molecular players during aneural AChR clustering compared to post-natal NMJ formation/stabilization.

Overall, these observations suggest that the loss of embryonic Ca_V_β1 isoforms during early post-natal stages significantly impacts endplate morphology without inducing major abnormalities in the expression of key synaptic proteins involved in its formation, nor perturbation of myofiber maturation.

### 3.5. Role of Embryonic Ca_V_β1 Isoforms in NMJ Maintenance in Adult Skeletal Muscle

**Figure 5:**
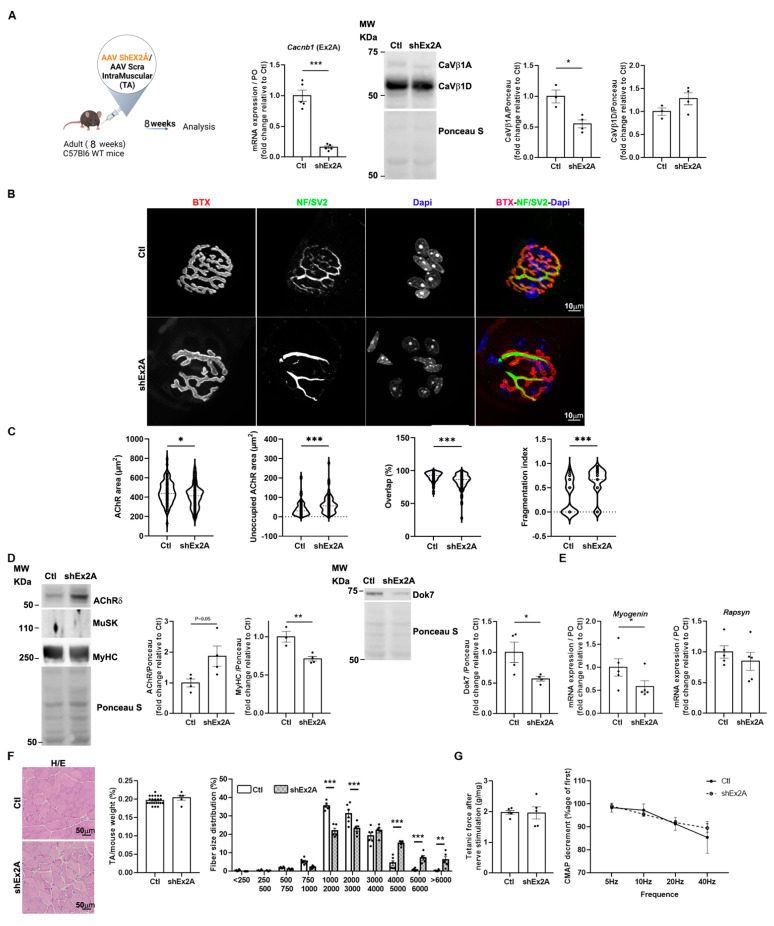
NMJ transcriptional and functional defects upon downregulation of embryonic Ca_V_β1 isoforms in adult TA muscles. (**A**) Schematic representation of the experimental protocol. RT-qPCR of embryonic *Cacnb1* variants using primer in exon2A in the control and shEx2A-treated TA muscles, Western Blot, and quantification of Ca_V_β1A and Ca_V_β1D in muscles 12 weeks post-injection. (**B**) Immunofluorescence (IF) images of the control and shEx2-treated TA muscles, 12 weeks post-injection, stained with α-bungarotoxin (BTX) for AChR (red), neurofilament light chain and Synaptic Vesicle Glycoprotein 2 (NF/SV2) (green), and DAPI (blue). (**C**) Quantification of NMJ morphology with area of AChR, unoccupied AChR area, overlap between nerve terminals and postsynaptic apparatus, and fragmentation index. (**D**) Western Blot and quantification of AChRδ, MuSK, and MyHC of Dok7, in the control and shEx2A-treated TA muscles 12 weeks post-injection. (**E**) RT-qPCR of *Myogenin* and *Rapsyn* in the control and shEx2A-treated muscles 12 weeks post-injection. (**F**) Hematoxylin and eosin (H/E) staining of TA, muscle weight, and cross-sectional area distribution (%) in the control and shEx2A conditions. Bar 50 μm. (**G**) Indirect (nerve) or direct (muscle) tetanic force measured in the control and shEx2A-treated TA adult muscles, 12 weeks post-injection. CMAP decrement measured by ENMG in the control and shEx2A-treated TA adult muscles, 12 weeks post-injection. All data are mean ± SEM (* *p* < 0.05, ** *p* < 0.01, and *** *p* < 0.001). *p* values were calculated by paired (A—Cacnb1 RT-qPCR-, E, F, G muscle weight) or unpaired *t*-tests (A—Ca_V_β1A and Ca_V_β1D WB-, C, D) and ordinary two-way ANOVA followed by Sidak’s multiple comparison test (G, CSA distribution).

To better understand if Ca_V_β1A and Ca_V_β1E isoforms may play a role in the stabilization of nerve–muscle synapses once they are completely formed and mature, we disrupted their expression in the TA of adult mice via the AAV-shEx2A injection (Figure 5A). We then analyzed morphological, molecular, and functional readouts reflecting NMJ integrity 12 weeks post-injection. The quantification of pre- and postsynaptic morphological parameters revealed that the perimeters and areas of both AChR and endplates were significantly reduced upon embryonic Cacnb1 depletion compared to the controls. Additionally, the size of synaptic contact and the overlap between nerve terminals and the postsynaptic apparatus were decreased. Furthermore, a significant NMJ fragmentation was observed upon downregulation of embryonic Ca_V_β1 proteins (Figure 5B,C).

Analysis of molecular players involved in NMJ formation and maintenance highlighted a significant increase in AChRδ protein expression, whereas the expression of MuSK protein was undetectable in all conditions (Figure 5D). In addition, we observed a significant decrease in Dok7 and MyHC protein expression (Figure 5D), and a decrease in *Myogenin* transcript levels, while those of *Rapsyn* were unchanged (Figure 5E). Next, we analyzed muscle histology, weight, and cross-sectional area (CSA) of treated muscles. We found no gross modifications of muscle histology, nor differences in muscle weight of embryonic Cacnb1-depleted TAs compared to the controls. However, CSA distribution showed a shift in the percentage of medium-sized area fibers toward larger-sized area fibers (Figure 5F), indicating a more subtle modification of muscle structure in the absence of embryonic Ca_V_β1 proteins. Finally, we measured muscle function in terms of force generation, by direct (muscle) and indirect (nerve) stimulation, and the neuromuscular connectivity by electroneuromyogram (ENMG) measurement of compound muscle activity potential (CMAP) decrement. We found that the ablation of embryonic Ca_V_β1 isoforms did not affect all these parameters, indicating that NMJ morphological alterations had no impact on muscle function, at least in that observed time window (Figure 5G).

Altogether, these data suggest that embryonic/perinatal Ca_V_β1 isoforms are (1) likely involved in NMJ formation prior to innervation, as supported by the in vitro study, (2) involved in the maintenance and/or maturation of the postsynaptic apparatus at early post-natal stages, and (3) involved in NMJ maintenance in adult muscles.

## 4. Discussion

The neuromuscular system plays a vital role for all vertebrates. Its proper development, maturation, and maintenance are strictly regulated processes requiring a multitude of mechanisms not yet completely elucidated. In this context, some years ago, a study reported that a component of ECC machinery, Ca_V_β1, the regulatory subunit of muscle voltage-gated Ca^2+^ channel encoded by the *Cacnb1* gene, was essential in the formation of nerve–muscle pre-patterning during embryonic development [5]. In a further study, this protein was demonstrated as having transcription factor properties regulating myogenic factors in muscle precursor cells [3]. More recently, two Ca_V_β1 skeletal muscle isoforms were identified by our work, Ca_V_β1D, defined as the adult constitutive isoform, and Ca_V_β1E, defined as an embryonic isoform with a role in up-regulating the expression of *Gdf5* and thus limiting muscle mass loss after denervation and during aging [1].

Here, we aimed to further characterize Ca_V_β1 isoforms by investigating the mechanisms behind the regulation of their expression and deciphering their role in the formation, maturation, and stability of the neuromuscular system. The use of Nanopore sequencing as an innovative and powerful exploration technology allowed the identification of *Cacnb1A*, coding for Ca_V_β1A protein, an additional embryonic isoform, expressed in adult muscle after denervation. Investigating the mechanisms regulating the differential expression of *Cacnb1* transcripts, we discovered that adult *Cacnb1D* and embryonic/perinatal *Cacnb1A* and *Cacnb1E* are expressed through the activation of two distinct promoters located in exon1 (Prom1) and exon2B (Prom2), which are differently regulated during development. Indeed, we have demonstrated that Prom1 is active during embryogenesis, while Prom2 is active in mature adult muscle, perinatal stages marking a period of transition between the activation of these two promoters. In addition, we have shown that, in adult muscle, nerve damage leads to the restoration of the embryonic epigenetic program of *Cacnb1* promoters. This evidence sheds light on the molecular events behind Ca_V_β1 modulation and presents new hypotheses on other upstream factors potentially implicated in the fine-tuning of their epigenetic program.

To gain insights into transcription factors binding at these promoter regions and potentially regulating them, we used the UCSC Genome Browser, an online software allowing comprehensive visualization and analysis of genomic data, including features like transcription factor binding sites. Among the wide range of transcription factors potentially binding to Prom1 and Prom2 regions, we identified Myogenin, a muscle-specific factor known to promote transcription of genes significant for myogenesis and AChR clustering [26,27]. In addition, we found, as potential binding factors of the Prom2 region, BRD4 and MEF2A, which are known to act as regulators of catabolic and myogenesis-related genes [28,29] and control muscle gene transcription [30], respectively. On the other hand, we identified, as potentially binding Prom1, KDM1A (or LSD1), which is also described to regulate key myogenic transcription factors and modulate muscle regeneration and recovery after injury [31]. More investigations are needed to define molecular interactors affecting the expression of *Cacnb1* variants; however, the data presented in this study pinpointed some possible players having a role in this event. Among these factors, we consider Myogenin and Mef2a as the most interesting candidates, inducing the use of the alternative promoter. Indeed, their expression increases following denervation [32], which could affect both Cacnb1 Prom1 activation and Prom2 inhibition.

Overall, the existence of various data Ca_V_β1 variants raises further questions about the significance of multiple isoforms during development and for skeletal muscle homeostasis, especially in a VGCC-independent context. We thus investigated the role of Ca_V_β1 on the different stages of NMJ formation, maturation, and stability. As mentioned, a previous study demonstrated that genetically ablated *Cacnb1* mice exhibited muscle patterning defects and aberrant innervation at E14.5 and E18.5 [5]. However, these alterations were observed after motor growth cones had already reached the muscle. To better understand the potential role of CaVβ1 isoforms in AChR pre-patterning *prior* to innervation—a gap that remains poorly explored in the literature—we investigated this process using an in vitro myotube model.

We found an increased size of aneural AChR clusters associated with precocious myotube maturation, which aligns with Chen and colleagues’ post-innervation results that showed increased endplate areas and more perforated clusters, indicative of precocious maturation. Our findings suggest that the defects observed in their study originate from abnormalities occurring before innervation begins. However, it cannot be excluded that these alterations could have been modified or amplified by nerve-derived factors.

The observed rise in AChR cluster size may be the consequence of different outcomes: i) the aberrant myotube maturation that could affect cluster enlargement due to increased myotube width; ii) the increase in MuSK level, possibly resulting from a transcriptional effect of myogenin [27], which may be connected with the expanded AChR clusters size [33]; and iii) the aberrant AChR aggregate stabilization due to increased Rapsyn levels [23,24]. Further analyses would be needed to elucidate all the mechanisms governed by Ca_V_β1A and Ca_V_β1E and implicated in the orchestration of AChR pre-patterning. However, this study indicates a crucial involvement of these proteins in the first steps of NMJ formation, independently of innervation and ECC modulation.

Going further in exploring the function of Ca_V_β1A/E isoforms on endplate maturation/stability during early post-natal life, we found that ablation of these proteins in the muscle of 1-month-old pups affected NMJ structure. The observed defects could be due to alterations in maturation, stability, or a combination of both, and distinguishing between these two processes is a challenging task. Regarding the possible cause of the observed defects, the increased muscle maturation occurring under ablation of the embryonic/perinatal Ca_V_β1 isoforms could lead to NMJ fragmentation as a mechanism to extend the AChR area following precocious muscle growth. Indeed, the link between NMJ fragmentation and degeneration might not be systematic, and some studies suggest that this event could also be a sign of regeneration, as a process of synaptic plasticity aimed at enlarging the synaptic area [34,35,36].

In adult muscle, despite the apparently weak expression of Ca_V_β1A/E, its ablation leads to significant alteration of NMJ morphology. Noteworthy, similar features are observed in pathophysiological conditions as during aging [1]. Indeed, age-related morphological defects of endplates, including postsynaptic fragmentation, reduced AChR density associated with synaptic decoupling, are reported as preceding changes in NMJ molecular marker expression [12,37]. This appears to be what we observed upon Ca_V_β1A/E ablation with no global modification in NMJ-associated actors despite morphological alterations. It is thus conceivable that functional deficits, associated with major alterations in NMJ-related signaling, would appear much later than the beginning of Ca_V_β1A/E downregulation. Deeper analyses will be needed to validate this hypothesis and elucidate the relationship between the expression levels of Ca_V_β1 variants in adult healthy muscle and an aging phenotype in mice, as suggested by our findings.

## 5. Conclusions

Taken together, our study sheds light on the effects of both Ca_V_β1A and Ca_V_β1E isoforms on distinct NMJ-specific stages. It would be of high relevance in future investigations to clarify whether one or both Ca_V_β1A and Ca_V_β1E isoforms are responsible for the observed effects.

In conclusion, in this work, we identified Ca_V_β1A as a novel Ca_V_β1 isoform and highlighted molecular and functional aspects of the different Ca_V_β1s as molecular players involved in the neuromuscular system formation, maturation, and maintenance.

## Data Availability

The Oxford Nanopore data were analyzed as previously described [15], and Fastq files generated in this study were deposited in the ENA database (https://www.ebi.ac.uk/ena/browser; access date 6 December 2025) under accession code PRJEB89914. Other materials are available upon request.

## Supplementary Materials

The following are available online at https://www.mdpi.com/article/10.3390/cells14151210/s1: Figure S1: Cacnb1 exon2B is systematically excluded when exon1 is expressed; Figure S2: Two specific and distinct promoters drive the expression of Cacnb1 isoforms in skeletal muscle; Table S1: Ir ab; Table S2: IIr ab; Table S3: Primers; Table S4: Primers Nanopore.
