# Supplementary material for: Identification of CaVβ1 Isoforms Required for Neuromuscular Junction Formation and Maintenance
Source: Cells. 2025 Aug 6;14(15):1210. doi: 10.3390/cells14151210 (PMC12346070; doi:10.3390/cells14151210)
Supplement: Supplementary file 1 [file cells-14-01210-s001.zip › cells-3780374-supplementary figues.pdf]

A

Ex1 Ex2A Ex2B Ex3

	TA inn	TA den	GAS inn	GAS den	Somme
	100,00	100,00	100,00	100,00	100,00
11000111111111111	93,84	90,44	71,13	88,05	85,86
11000110000000001	0,00	0,00	10,43	0,10	2,63
11000111111011111	1,86	1,92	1,59	1,82	1,80
11000000000000011	0,00	0,01	6,72	0,06	1,70
11000110000000111	0,00	2,01	4,59	0,01	1,65
11000111101111111	0,78	1,26	0,62	0,97	0,91
11000100000000001	0,01	0,00	0,49	2,81	0,83
11000111110111111	0,61	0,78	0,68	0,85	0,73
11000111011111111	0,74	0,66	0,57	0,74	0,68
10000111111111111	0,62	0,43	0,49	0,49	0,51
11000111111111011	0,36	0,25	0,28	0,43	0,33
10000000000000001	0,26	0,54	0,35	0,11	0,31
11000100000000011	0,00	0,00	0,01	1,11	0,28
10000000000000011	0,00	0,00	0,69	0,02	0,18
11000111110000011	0,00	0,00	0,00	0,66	0,16
11000011111111111	0,14	0,16	0,15	0,19	0,16
11000110000000011	0,00	0,02	0,04	0,55	0,15
11000100011111111	0,00	0,01	0,52	0,01	0,14
11000111110011111	0,15	0,11	0,08	0,14	0,12
10000000000001111	0,00	0,41	0,01	0,00	0,10
11000111111101111	0,11	0,10	0,08	0,11	0,10
11000101111111111	0,09	0,12	0,09	0,11	0,10
11000110111111111	0,06	0,05	0,06	0,08	0,06
11000100000000111	0,00	0,22	0,01	0,00	0,06
11000110011111111	0,05	0,05	0,01	0,08	0,05
10000111111011111	0,05	0,06	0,03	0,02	0,04
11000111111001111	0,06	0,03	0,03	0,03	0,04
11000111011011111	0,04	0,03	0,02	0,04	0,03
11000111100111111	0,04	0,02	0,05	0,02	0,03
11000111111110111	0,02	0,04	0,03	0,04	0,03

0 = Exon absent / 1 = Exon present

B

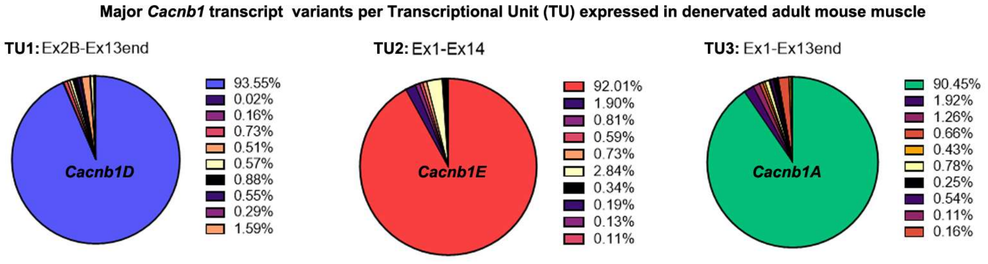

Figure S1. *Cacnb1* exon2B is systematically excluded when exon1 is expressed. A. Long-read Nanopore sequencing data depicting exon presence and absence for each *Cacnb1* variant (only the first 30 are shown) in innervated (inn) and denervated (den) mouse *Tibialis Anterior* (TA) and *Gastrocnemius* (GAS) muscles. Each row represents an individual transcript, with "1" indicating the presence and "0" indicating the absence of specific exons. This binary representation allows the observation that none of the transcripts starting at exon1 include exon2B, suggesting that alternative splicing is not the mechanism regulating exon2B inclusion but rather

indicates the existence of an alternative promoter at exon2B. **B.** Percentage of *Cacnb1* major isoform per TU after Nanopore sequencing in denervated muscle.

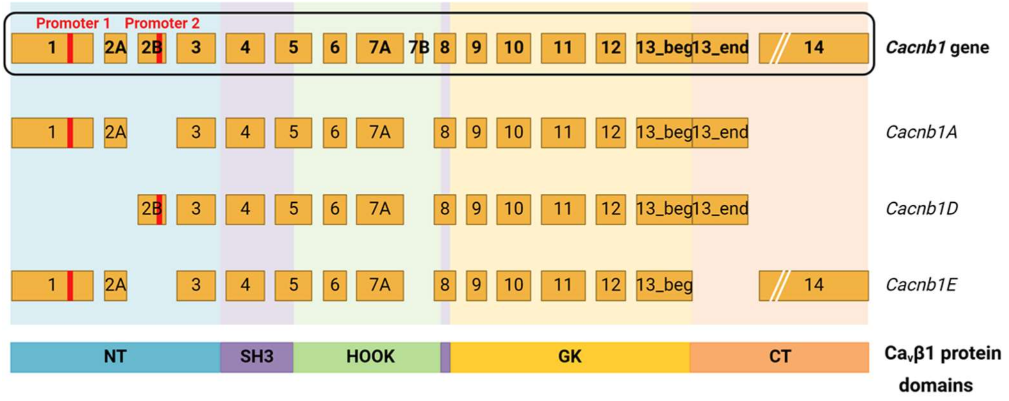

Figure S2. Two specific and distinct promoters drive the expression of *Cacnb1* isoforms in skeletal muscle. *Cacnb1* gene and skeletal muscle transcript variants: *Cacnb1A*, *Cacnb1D*, and *Cacnb1E*, and the corresponding promoter sites, as well as the corresponding protein domains, are represented.
